# Assessment of decisional capacity. A systematic review and analysis of instruments regarding their applicability to requests for assisted suicide

**DOI:** 10.1192/j.eurpsy.2025.10041

**Published:** 2025-07-08

**Authors:** Leonie Kupsch, Jakov Gather, Jochen Vollmann, Stephan Nadolny, Jan Schildmann

**Affiliations:** 1 Institute for History and Ethics of Medicine, Interdisciplinary Centre for Health Sciences, Medical Faculty of Martin Luther University Halle-Wittenberg, Halle (Saale), Germany; 2 Institute for Medical Ethics and History of Medicine, Ruhr University Bochum, Bochum, Germany; 3 Department of Psychiatry, Psychotherapy and Preventive Medicine, LWL University Hospital, Ruhr University Bochum, Bochum, Germany

**Keywords:** assisted suicide, decisional capacity, instruments, systematic review

## Abstract

**Background:**

Decisional capacity is an important requirement for assisted suicide, which has been legalized in an increasing number of countries. While several instruments have been developed over the past few decades to assess the capacity to consent to treatment, little is known about their applicability to assessing capacity in the context of requests for assisted suicide.

**Methods:**

Systematic review of instruments assessing decisional capacity published up to March 2024. Data concerning criteria for determining decisional capacity, psychometric properties, and other aspects were extracted from all instruments included. Selected instruments were analyzed regarding their applicability to requests for assisted suicide.

**Results:**

We identified 23 instruments assessing the capacity to consent to treatment. There is considerable heterogeneity regarding the criteria utilized for assessing decisional capacity and their operationalization. Next to more cognitive abilities, some instruments directly incorporated emotions and values. Five instruments were assessed for applicability to requests for assisted suicide. The framing of decisional capacity within the context of disease and treatment options frequently limits the application of instruments to assess decisional capacity in the context of requests for assisted suicide.

**Conclusions:**

No instrument could be identified that could be applied to assessing decisional capacity in the context of requests for assisted suicide without any limitations or without necessitating adjustments. Further normative and empirical work is required for developing an instrument that could be applicable in this context.

## Introduction

Assisted suicide refers to assisting a person in ending their own life, with the final action being carried out by the person themselves. This practice has been legalized in an increasing number of countries [1, 2]. Decisional capacity is an important requirement for legally and ethically justified assisted suicide [3–6]. If a person is of legal age, they are generally assumed to have decisional capacity for decision-making situations regarding medical treatment. A formal assessment of decisional capacity should be conducted for a specific situation and context only if there are any reasons for doubts [7–9]. By contrast, a proactive formal assessment of decisional capacity or a reliable elimination of aspects that could impair decisional capacity is often called for in the context of requests for assisted suicide [10–13].

Different terms including “capacity,” “competence,” and “competency” are mentioned when referring to decisional capacity, which is partly due to linguistic or conceptual differences. For this article, these terms were subsumed under “decisional capacity.” A widely used definition of decisional capacity includes four abilities as outlined by Grisso and Appelbaum [7, 14]:

1. The ability to understand information relevant to the decision at hand (*understanding*)
2. The ability to apply relevant information to one’s own situation in the sense of acknowledging one’s illness and treatment options (*appreciation*)
3. The ability to weigh and evaluate options regarding possible consequences in a logically consistent way (*reasoning*)
4. The ability to *evidence a choice*

Some authors have criticized this definition of decisional capacity and its focus on cognitive abilities as insufficient. They call particularly for the inclusion of values, emotions, and social aspects to ascertain decisional capacity [8, 15].

Empirical studies indicate that in practice, many clinicians do not refer to standardized procedures or specifications but rather take an individual approach to assessing decisional capacity [4, 5, 16]. This can lead to heterogenous results depending on the clinician doing the assessment [17]. Furthermore, the personal values of clinicians could potentially influence capacity assessment [10, 18]. Against this background, establishing a procedure with predefined criteria could help to support the process of assessing decisional capacity. This seems particularly needed in far-reaching decisions, such as in the context of requests for assisted suicide.

A number of instruments assessing capacity to consent to treatment have been developed in the past few decades [19–23]. However, little is known about their applicability to assessing decisional capacity in cases of requests for assisted suicide, as these cases do not necessarily constitute a medical decision-making situation. The aim of this paper is, firstly, to present and appraise published instruments assessing capacity to consent to treatment based on a systematic literature review. Secondly, all instruments assessing decisional capacity by including all four abilities outlined by Grisso and Appelbaum [7] and in relation to a specific decision were analyzed regarding their applicability to requests for assisted suicide.

## Methods

### Systematic review

Based on a recent systematic review by Amaral et al. [19] on instruments assessing capacity to consent to treatment published up to 2018, we conducted a search for instruments published since 2018 in relevant databases (MEDLINE [Ovid], Web of Science, PsycINFO, CINAHL, Cochrane Central Register of Controlled Trials [CENTRAL]) using a modified search strategy on October 1, 2022, and again on March 15, 2024. Search strategies and respective results are documented in Appendix 1 (Supplemental material 1).

Criteria for inclusion and exclusion of articles were modeled on the approach of Amaral et al. [19]: Instruments assessing capacity to consent to treatment were included, while those assessing capacity to consent to research, create advance directives, or for non-medical decisions were excluded. Furthermore, all instruments assessing decisional capacity in children and adolescents, guidelines on decisional capacity, and instruments without specifications for practical application were also excluded.

**Figure 1.**
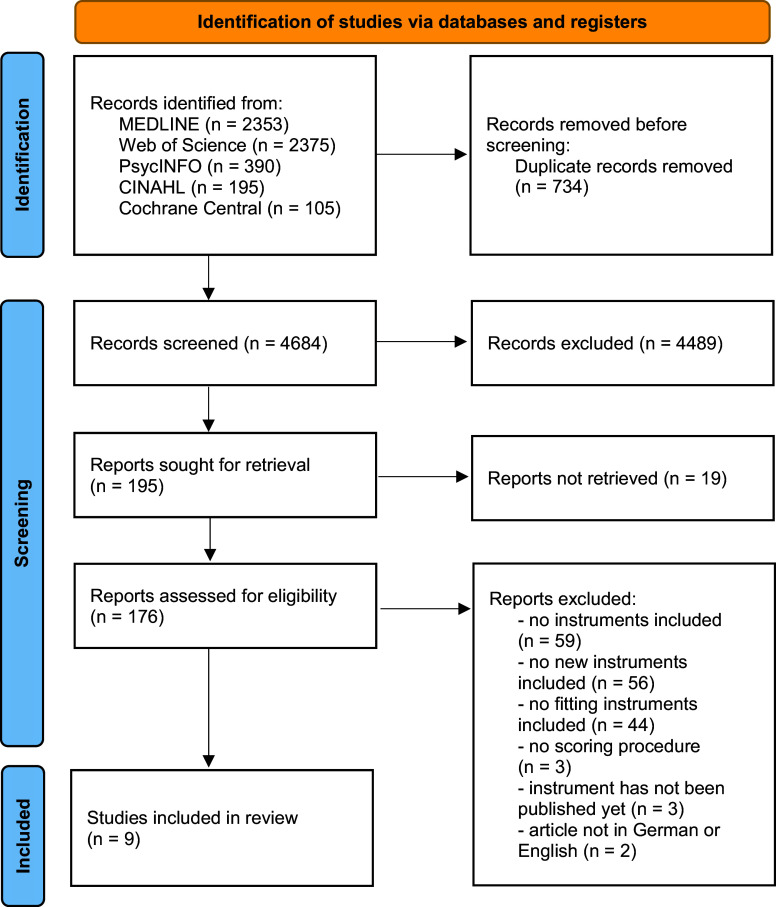
PRISMA 2020 flowchart.

After removing any duplicates, the initial search yielded 3628 articles that were then imported onto the platform Rayyan [24]. During the search update, 1056 additional articles published since October 2022 were identified. The titles and abstracts of all 4684 articles were screened and assessed independently by two researchers utilizing the predefined criteria. Potentially relevant articles identified in this way were included or excluded based on their full text. Figure 1 documents the search and the screening process using the PRISMA 2020 flowchart [25].

Due to limited resources and the small number of newly included articles, no quality appraisal was performed. Data on various characteristics of the instruments were extracted from all articles identified by Amaral et al. [19] as well as from all newly included publications and summarized in Table 1.

**Table 1.** Overview of instruments assessing capacity to consent to treatment

Instrument/ publication/country	Format	Abilities assessed	Duration of use	Scoring procedure	Cut-off score	Psychometric properties (selection)	Pilot study population	Decisional capacity/further aspects
Aid to Capacity Evaluation (ACE) Etchells et al. 1999 [26], Etchells (ACE [27]) *Canada*	Semi-structured interview on the current treatment decision	Understanding, appreciation, (reasoning)	10–20 min	Adequacy of answers scored with yes/uncertain/no, no points awarded, overall assessment of decisional capacity	Not specified	Interrater reliability: 93% agreement, *κ* = 0.79 [95% CI 0.63, *n*/a] Agreement with experts at 0.90–0.95	Patients (*n* = 100) with pending decisions regarding a medical procedure or treatment	Assessment of decisional capacity for the decision at hand
Assessment of Capacity to Consent to Treatment (ACCT) Moye et al. 2007 [28] *USA*	Semi-structured interview on the current treatment decision or alternatively utilizing hypothetical vignettes	Understanding, appreciation, reasoning, evidencing a choice	Not specified	Points awarded for correct or accurate answers, total scores calculated for understanding, appreciation and reasoning, overall assessment of decisional capacity	Yes, two standard deviations below the control group mean	Interrater reliability: *r* = 0.90 Internal consistency: Cronbach’s *α* = 0.96 based on all capacity items across all three vignettes Agreement with clinicians at 82% (*κ* = 0.44)	Patients with dementia (*n* = 20), schizophrenia (*n* = 20) and without cognitive impairment (*n* = 19, control group)	Assessment of decisional capacity for the decision at hand or for hypothetical decision-making situations Additional assessment of values and social aspects as part of the instrument, including visual aids
Assessment of Consent Capacity - Treatment (ACC-T) Cea and Fisher 2003 [29] *USA*	Structured interview on three standardized treatment vignettes	Understanding, appreciation, reasoning, evidencing a choice	45 min	Points awarded for complete/correct answers, 0–2 points possible per question	Not specified	Interrater reliability: agreement at 97–98% Internal consistency: Cronbach’s α at 0.82–0.88 across all vignettes	People with mild (*n* = 30) and moderate learning difficulties (*n* = 30) and control group (*n* = 30)	Assessment of decisional capacity for hypothetical decision-making situations
Bedside Capacity Assessment Tool (BCAT) Carney et al. 2018 [30] *USA*	Semi-structured interview on the current (treatment) decision	Understanding, appreciation, reasoning, evidencing a choice	Not specified	Closed questions with answer options “yes” or “no/not sure,” no points awarded, overall assessment of decisional capacity	Yes, all four abilities must be present	Agreement with experts at 76.1%	Clinicians (*n* = 30) from various specialties (geriatrics, palliative medicine, internal medicine), application of BCAT to case vignettes	Assessment of decisional capacity for the decision at hand Further development of the CAT (see below) Reasoning should be set in relation to personal, cultural and religious values Not recommended for more complex cases, refer to psychiatric expertise or MacCAT-T
Capacity Assessment Tool (CAT) Carney et al. 2001 [31] *USA*	Semi-structured interview on the current (treatment) decision	Understanding, reasoning, evidencing a choice, (appreciation)	“Minutes,” not further specified	Points awarded for suitable/plausible answers in six categories, 0–1 or 0–3 points possible in each category	A specific score must be achieved in each category	Agreement with psychiatric evaluation at 80–100% (κ = 0.58–1.00)	Patients (*n* = 20; recruited from geriatric ward or via consultation service) with pending decisions regarding a medical procedure or treatment	Assessment of decisional capacity for the decision at hand Additional assessment of communication skills and orientation to person, place and time
Capacity to Consent to Treatment Instrument (CCTI) Marson et al. 1995 [32], Gerstenecker et al. 2016 [33] *USA*	Structured interview on two case vignettes	Understanding, appreciation, reasoning, evidencing a choice	20–25 min	Points awarded for correct answers in five categories, 0–2 points possible in each category	Yes, two standard deviations below the control group mean	Interrater reliability: *r* > 0.83	Patients with mild Alzheimer’s dementia (*n* = 15) and patients with moderate Alzheimer’s dementia (*n* = 14) and control group (*n* = 15)	Assessment of decisional capacity for hypothetical decision-making situations Age-independent and age-adjusted normative data available
Competency Interview Schedule (CIS) Bean et al. 1994 [34] *Canada*	Structured interview on the decision for/against electroconvulsive therapy (ECT)	Understanding, appreciation, reasoning, and evidencing a choice	Not specified	Rating of responses on a 7-point Likert scale; 1–3 for adequate, 4 for marginal and 5–7 for inadequate responses; calculation of a total score not recommended	Not specified	Interrater reliability: ICC = 0.95 Retest reliability: *r* = 0.79 Internal consistency: Cronbach’s α =0.96 Correlation with assessment by clinicians at 0.35–0.73	Patients (*n* = 96) in a psychiatric clinic with an indication for ECT	Assessment of decisional capacity specifically for electroconvulsive therapy
Compulsory Assessment and Treatment - Capacity Assessment Tool (CAT-CAT) Kumar et al. 2022 [35] *Australia, New Zealand*	Semi-structured interview on a comic sequence and the current treatment decision in the context of a substance use disorder	Understanding, evidencing a choice, retention, (appreciation, reasoning)	15–20 min	Points awarded for correct answers, calculation of total score for subtests and for CAT-CAT	Yes, at least 50% of all points must be achieved in each subtest	Interrater reliability: ICC = 0.998 Retest reliability: ICC = 0.996 overall; understanding: ICC = 0.790 [95% CI 0.117; 0.948]; evidencing a choice: ICC = –0.354,*κ* = 0.538 [95% CI 0.240; 0.836]	Practitioners (*n* = 13): first training, then application of the CAT-CAT to two practice cases; additionally people without substance use disorder (*n* = 35)	Assessment of decisional capacity specifically for treatment for substance use disorder
Decision Assessment Measure Wong et al. 2000 [36] *England, Wales*	Semi-structured interview on the decision for/against a blood test	Understanding, evidencing a choice, retention	Not specified	Points awarded for correct answers, 0–2 points possible per question	Not specified	Interrater reliability: *κ* = 0.87	Patients (*n* = 21) with schizophrenia or schizoaffective disorder, patients (*n* = 21) with dementia, people with learning difficulties (*n* = 20), control group (*n* = 20)	Assessment of decisional capacity specifically for blood tests Additional non-verbal assessment of decisional capacity via a demonstration of the information conveyed
Direct Assessment of Decision-Making Capacity Fitten and Waite 1990 [37] *USA*	Structured interview on three case vignettes	Understanding, reasoning	Not specified	Points awarded for complete answers, 0–2 points possible per question	Yes, total score below the lower 99.5% CI of the control group mean	Agreement with doctors at 72%	Hospitalized patients (*n* = 25) with acute illness, without neurological/psychiatric illness and control group (*n* = 25, matched for age and education)	Assessment of decisional capacity for hypothetical decision-making situations
Dundrum Capacity Ladders (DCL) Moynihan et al. 2018 [38] *Ireland*	Semi-structured interview (“ladders”), either on three case vignettes or on the current decision	Understanding, appreciation, reasoning, and evidencing a choice	Not specified	Points awarded for each of the four abilities, 0–100 points possible for each, total score is calculated	Not specified	Interrater reliability: ICC = 0.995 Internal consistency: Cronbach’s α at 0.960–0.973 across all three vignettes	Patients with schizophrenia (*n* = 48) and with schizoaffective disorder (*n* = 7)	Vignettes assess decisional capacity for hypothetical decision-making situations, but “ladders” can also be applied to current decision-making situation Vignettes on three domains (finances, welfare, healthcare)
Visual Decisional Aids Chang and Bourgeois 2020 [39] *USA*	Semi-structured interview on two case vignettes, with accompanying decisional aids	Understanding, appreciation, reasoning, evidencing a choice	No exact specifi-cation, <30–45 min	Points awarded for different aspects of the four abilities, points between 0–1 and 0–6 are possible	Not specified	Interrater reliability: agreement at 95–96.67%	Patients with mild (*n* = 11) and moderate dementia (*n* = 9)	Assessment of decisional capacity for hypothetical decision-making situations Visual decisional aids (images and text) were created for both vignettes
Hopemont Capacity Assessment Interview (HCAI) Edelstein 2000 [40] *USA*	Semi-structured interview on two case vignettes	Understanding, appreciation, evidencing a choice, (reasoning)	30–60 min	Points awarded for correct answers, 0–2 points possible per question	Not specified	Interrater reliability: *r* = 0.93 Retest reliability: *r* = 0.29 Internal consistency: Cronbach’s *α* = 0.94 (Dunn et al. 2006 [21])	No exact information, probably nursing home residents (Dunn et al. 2006 [21])	Assessment of decisional capacity for hypothetical decision-making situations Includes brief explanation of important concepts and assesses understanding
MacArthur Competence Assessment Tool-Treatment (MacCAT-T) Grisso et al. 1997 [41], Grisso and Appelbaum 1998 [7], Scholten and Haberstroh 2024 [14] *USA*	Semi-structured interview on the current treatment decision	Understanding, appreciation, reasoning, evidencing a choice	15–25 min	Points awarded for respective abilities, points between 0–2 and 0–8 are possible, calculation of a total score not recommended	No	Interrater reliability: ICC for skills at 0.87–0.99; ICC for individual items at 0.82–0.99	Patients with schizophrenia or schizoaffective disorder (*n* = 40) and control group (*n* = 40, matching with regard to key characteristics)	Assessment of decisional capacity for the decision at hand Validated for different groups of people and situations as well as in different languages
MacArthur Treatment Competence Study Instruments (UTD, POD, TRAT) Appelbaum and Grisso 1995 [42], Grisso and Appelbaum 1995a [43], Grisso and Appelbaum 1995b [44] *USA*	See below	Understanding, appreciation, reasoning, evidencing a choice	60–90 min	Scores of the individual instruments are not added together	Not specified	See below	Patients with schizophrenia or schizoaffective disorder (*n* = 75), patients with major depressive disorder (*n* = 92), medical inpatients (*n* = 82); three control groups (same number of people and matching with regard to key characteristics)	See below
1. *Understanding Treatment Disclosures (UTD*)	Structured interview on one of three case vignettes	Understanding	25–30 min	Points awarded for correct answers, 0–2 points possible, total score not calculated, but calculation of scores for “subtests”	Not specified	Interrater reliability: ICC > 0.84 Internal consistency: Cronbach’s *α* = 0.55–0.85 Retest reliability: *r* = 0.30–0.80	See above	Possibly assesses decisional capacity for the decision at hand as vignette was matched to the person’s illness
2. *Perceptions of Disorder (POD)*	Structured interview on the current illness	Appreciation	10–20 min	Person being assessed appraises information and its relevance for themselves on a 6-point Likert scale, points awarded by the assessor for correct answers, 0–2 points possible, total score not calculated	Not specified	Retest reliability: *r* = 0.48–0.90 Internal consistency: Cronbach’s *α* = 0.67–0.80	See above	Partial assessment of decisional capacity for the decision at hand as specific information on the person being assessed is included
3. *Thinking Rationally About Treatment (TRAT)*	Structured interview on one of three case vignettes and three tasks	Reasoning, evidencing a choice	25–30 min	Points awarded for completion of “subtests” for the vignette, 0–2 points possible for each subtest; 0–3 points awarded depending on the completion of the tasks	Not specified	Interrater reliability: ICC > 0.85 Retest reliability: *r* = 0.44–0.68 Internal consistency: Cronbach’s *α* = 0.39–0.74	See above	Assessment of decisional capacity for hypothetical decision-making situations
Mental Capacity Assessment Support Toolkit (MCAST) Jayes et al. 2021 [45], Jayes et al. 2022a [46], Jayes et al. 2022b [47] *England, Wales*	“Support Tool”: guideline for assessing decisional capacity for the decision at hand “Communication Screening Tool”: instrument for identifying difficulties in communication	Understanding, reasoning, evidencing a choice, retention	Not specified	Adequacy of answers scored with yes/no and sometimes also “uncertain,” no points awarded, free text answers, overall assessment of decisional capacity	Not specified	“Support Tool”: not specified “Communication Screening Tool”: Interrater reliability: *κ* = 0.432 [95% KI –0.053; 0.917] Criterion validity: *κ* = −0.370 [95% KI –0.882; 0.144]	Practitioners (*n* = 21) and patients with stroke (*n* = 7) and cognitive impairment (*n* = 10)	Assessment of decisional capacity for the decision at hand Toolkit also includes “Resource Pack” with (visual) materials to facilitate communication Supports the preparation, implementation and documentation of the capacity assessment Only available as a prototype to date
Structured Interview for Competency Incompetency Assessment Testing and Ranking Inventory (SICIATRI) Tomoda et al. 1997 [48] *Japan*	Structured interview on the current treatment decision	Understanding, evidencing a choice (appreciation), awareness regarding information and current decision-making process	20 min	Points awarded for correct answers, 1–3 points possible, overall assessment between level 0 (“completely incompetent”) and level 4 (“completely competent”)	Not specified	Interrater reliability: *κ* = 0.14–0.82 Agreement with clinicians at 81.3%	Patients (*n* = 25) with a psychiatric illness (*n* = 25) and medical inpatients (*n* = 23)	Assessment of decisional capacity for the decision at hand
Two-Part Consent Form Roth et al. 1982 [49] *USA*	Questionnaire completed by patients on the consent form on electroconvulsive therapy or on a study	Understanding	Not specified	Between 0 and 2 points awarded per question, calculation of total score	Not specified	Interrater reliability: *r* = 0.96 Retest reliability: *r* = 0.76	Patients (*n* = 57) with indication for ECT who had consented (*n* = 44) or not consented (*n* = 13) to ECT and control group (*n* = 44)	Only assesses understanding
Urteilsfähigkeit-Dokument (U-Doc) Hermann et al. 2020 [50], Thomas-Hund 2021 [51], Trachsel and Biller-Andorno 2022 [52] *Switzerland*	Semi-structured interview on the current treatment decision	Understanding, appreciation, reasoning, evidencing a choice	Up to 30 min	No points awarded, assessment of 12 criteria as “unaffected”/"impaired”/ “unclear” and three categories as “unaffected”/"slightly impaired”/"moderately impaired”/"strongly impaired”/"unclear”	No	Not specified	Qualitative study: workshop with multidisciplinary team on U-Doc, training of practitioners (*n* = 84) and 4 months of use in a clinical setting, then interviews with practitioners (*n* = 24)	Assessment of decisional capacity for the decision at hand Focuses on the assessment of values, emotions and biographical factors and risk factors for possible coercion Includes reflections on potential biases of the assessors
Vignette method by Schmand et al. Schmand et al. 1999 [53] *Netherlands*	Structured interview on two case vignettes	Understanding, appreciation, reasoning, evidencing a choice	Not specified	Points awarded for correct answers, scores calculated for each vignette, calculation of total score	Yes, 95% of the total score of the control group	Internal consistency: Cronbach’s *α* = 0.69–0.74 Agreement with clinicians at *κ* = 0.36	Patients (*n* = 64) with dementia (of which mild: *n* = 43, moderate: *n* = 7, minimal: *n* = 14) and control group (*n* = 176)	Assessment of decisional capacity for hypothetical decision-making situations
Vignette method by Vellinga et al. Vellinga et al. 2004 [54], Vellinga et al. 2005 [55] *Netherlands*	Structured interview on case vignettes	Understanding, appreciation, reasoning, evidencing a choice	Not specified	Points awarded for correct answers, 0–2 points possible, calculation of total score	Yes, 95% of the total score of people without cognitive impair-ments	Interrater reliability: *κ* = 0.64 Agreement at 78%	Geriatric patients with age over 65 (*n* = 80), of which *n* = 30 had dementia	Possibly assesses decisional capacity for the decision at hand as vignettes on the person’s illness were selected in some cases

### Structured analysis of the potential applicability of assessment instruments to requests for assisted suicide

After extracting any descriptive data, instruments were analyzed regarding their applicability in practice to requests for assisted suicide. We defined two inclusion criteria for this structured analysis: First, instruments should assess all four abilities outlined by Grisso and Appelbaum [7], as they are widely regarded to be relevant for assessing decisional capacity [8, 9]. Second, only instruments that can be used for assessing capacity for the decision at hand were included, as decisional capacity is generally interpreted as a context-specific ability [7–9]. Furthermore, the current and individual situation of the person making the request should be taken into account, particularly regarding the assessment of decisional capacity in the context of requests for assisted suicide.

All instruments fulfilling both inclusion criteria were then analyzed regarding their applicability to requests for assisted suicide using four questions that are relevant in the context of the normative and legal requirements for assessing decisional capacity in cases of requests for assisted suicide:

1. For which specific context was the instrument designed? Is **a decision-making situation regarding treatment options a premise of the instrument,** or is it possible to assess decisional capacity if there are **no treatment options** or **even in the absence of an underlying illness**?
2. Is the **scope of application** of the assessment instrument **limited to a specific illness or group of people,** or can it be **applied to different situations and groups of people**?
3. Does the instrument assess **other aspects** (e.g. values or emotions) **in addition to the four abilities** outlined by Grisso and Appelbaum?
4. Does the instrument **include information about the assessors**, for example, regarding reasons that could potentially influence or distort the assessment of decisional capacity?

The first two questions are based on the fact that reasons and motives for requesting assisted suicide can differ, and not every country allowing assisted suicide requires requestors to have some kind of underlying illness [1, 13]. As there is also a call for the inclusion of, for example, values and emotions to determine their possible impact on a person’s decisional capacity [8, 15], the third question analyzes whether aspects other than the four abilities are assessed. Lastly, the fourth question is based on studies showing that personal values and attitudes of the assessor regarding assisted suicide could possibly influence requirements and thresholds for decisional capacity [10, 15]. All four questions were developed for analyzing the assessment instruments regarding their applicability to requests for assisted suicide, but are not intended as set requirements.

## Results

We identified a total of 23 instruments assessing the capacity to consent to treatment. In addition to the 17 instruments [7, 14, 26–29, 31–34, 36, 37, 40–44, 48, 49, 53–55] Amaral et al. [19] had already found in the course of their review, 6 new instruments published since 2018 and reported in nine publications [30, 35, 38, 39, 45–47, 50, 52] were identified.

An overview of data extracted regarding the format of the instruments, abilities assessed by the instruments, duration of use, scoring procedures and cut-off scores, psychometric properties, and pilot study populations can be found in Table 1.

When comparing the various instruments, similarly to the general discussion, different terms are mentioned when referring to decisional capacity. In light of the core conceptual and empirical similarities between the instruments, only the term “decisional capacity” is used throughout this article, though it should be noted that the instruments primarily assess the abilities that are considered to be required for decisional capacity. Regarding the majority of instruments, their conceptualization of decisional capacity is based on the four abilities outlined by Grisso and Appelbaum [7]. However, there are different approaches to operationalizing these abilities.

### Operationalization of understanding, appreciation, reasoning, and evidencing a choice

Regarding **understanding**, instruments usually assess whether a person is able to *comprehend information related to their illness*, *possible treatment options*, and *risks and benefits.* Some instruments also include further aspects, such as determining whether the person being assessed understands that they can *refuse the proposed treatment options* [26, 49], what the *purpose* or *objective of the treatment* is [35–37], or what *risks and benefits* may result from *refusing treatment* [36, 48, 50].

There are two different methods being primarily used to operationalize **appreciation**: Some of the instruments assess appreciation as the *acknowledgement of illness and possible treatment options* [7, 38–40, 42–44]. Other instruments determine whether the *potential consequences of a decision* can be *anticipated* [26, 29, 32, 53]. Several instruments [28, 30, 34, 50, 54] utilize both methods. Another aspect that is sometimes included in the assessment of appreciation is a possible distrust of medical staff [28, 34].

**Reasoning** is defined as the *ability to manipulate information rationally* by *comparing, weighing*, and *evaluating options logically and consistently*, especially concerning *possible consequences and effects on one’s own life situation*, which is operationalized in this way by some instruments [7, 31, 39, 42–44, 54]. Other instruments focus on *weighing risks and benefits* [28, 29] or relevant information in general [46, 50]. The *logically consistent justification of the decision* [30, 32, 34, 50, 53] is an important aspect of the assessment of reasoning for some instruments.

Assessing the ability to **evidence a choice** includes a direct question about the final decision of the person being assessed for the majority of the instruments characterized. In some cases, it should also be documented whether the person would like to *make the decision themselves or rather have someone else decide for them* [34, 48]. One instrument also emphasizes aspects such as *communication and orientation* as part of the assessment [31].

### Possible applicability in the context of requests for assisted suicide

Applying the inclusion criteria outlined under 2.2, six instruments [7, 28, 30, 34, 38, 50] were analyzed regarding their possible applicability to assessing decisional capacity in the context of requests for assisted suicide, utilizing the four questions for structured analysis.

The results of the analysis for five of the six instruments are described below. An analysis of the Dundrum Capacity Ladders [38] was not possible as the instrument was not available during research or after contacting the authors.

#### Assessment of Capacity to Consent to Treatment (ACCT [28])

The ACCT is based on the premise that the person being assessed has an illness and has to **make a decision regarding treatment options**, which is why, for example, for assessing understanding, information about the illness is provided followed by questions checking a person’s comprehension of their “medical problem” [28]. For the same reason, all questions aim at assessing the **capacity to consent to treatment** specifically. In order to assess whether a person acknowledges potential treatment options, for instance, they are asked, *inter alia*, if there are any doubts about the possible effectiveness of the proposed treatment or concerns about the doctors’ intentions. The assessors are given **specific questions** by the ACCT, but these can be **flexibly adapted** to a **variety of situations**. A special feature is the **additional assessment of social aspects and values,** including visual aids. **Information on assessors,** on the other hand, is **not** included.

#### Bedside Capacity Assessment Tool (BCAT [30])

The BCAT was also designed with a **decision-making situation regarding treatment options** in mind, which is why, for understanding, a person’s knowledge about their treatment choices or about their medical decision-making situation in general is assessed. The instrument is also based on the assumption that a person has an underlying illness. The **wording of questions** for assessing decisional capacity is **not specified**. Instead, different aspects required for decisional capacity are listed. Overall, the BCAT can be used for a **wide range of clinical situations**. It should be noted though that, according to the authors of the BCAT, the instrument is **not** intended **for use in more complex cases.** Assessors should check whether the reasons delineated are **consistent with values** held by the person being assessed. **Information relating to the assessors** is **not included**.

#### Competency Interview Schedule (CIS [34])

The CIS was created as a structured interview for patients with a **psychiatric illness** and an indication for **electroconvulsive therapy**. This is also reflected by the questions, for which **specific wording** is provided, and explicitly tests the decisional capacity for this intervention. Accordingly, there are limitations regarding the application for assessing decisional capacity for assisted suicide, as evidenced, for example, by the questions on whether the person believes that the medical staff expect them to remain in hospital or if the person wants the decision to be made by someone else. In addition to the four abilities outlined by Grisso and Appelbaum [7], various **other aspects** are **assessed**, such as whether a person feels **pressured or coerced into making a decision**. **Information on assessors** is **not part of the assessment**.

#### MacArthur Competence Assessment Tool – Treatment (MacCAT-T [7, 14, 41])

The MacCAT-T is also based on the **premise** that the person being assessed is affected by an illness and **has to make a decision regarding treatment options.** Examples include questions on acknowledging their illness or treatment options for assessing appreciation, or their comprehension of these treatment options, for which they should recount their own understanding of the treatment suggested. The MacCAT-T has already been used to assess decisional capacity for **various groups of people** [56, 57], and the **questions** can, therefore, be **flexibly adapted to many situations**. **Translations into different languages** are available. **Aspects other than the four abilities** outlined by Grisso and Appelbaum [7] and **information on the assessors** are **not included**.

#### Urteilsfähigkeit-Dokument (U-Doc [50–52])

The U-Doc was developed to assess decisional capacity in the context of the legal framework in Switzerland. While there are some terminological and conceptual differences due to this framework, all four abilities are incorporated into the instrument. Similar to the other instruments, a **decision-making situation regarding treatment options is also a premise** for the U-Doc. The latter form contains both suggested wording for questions and explanations of the abilities required for decisional capacity. Overall, the **wording** can be **flexibly adapted to the situation at hand** as necessary. As with the instruments discussed previously, the applicability to assessing decisional capacity for people with no underlying illness or suggested treatment options is limited, as the U-Doc mainly assesses appreciation as an acknowledgement of illness and treatment options, but it is **possible to directly adapt** some of the items **to non-medical decision-making situations**. In addition to the four abilities, the U-Doc also directly assesses other aspects, such as **values, biographical factors and emotions,** and the possible influence of **internal and external pressure** on decision-making. Furthermore, the form (which needs to be distinguished from the underlying concept) also includes a reminder that assessors should critically examine their judgment and **reflect on** their own **bias regarding personal values and conflicts of interest**.

Table 2 provides an overview of the structured analysis of the instruments using the predefined criteria.

**Table 2.** Structured analysis of the possible applicability of instruments assessing decisional capacity to requests for assisted suicide

Questions for structured analysis	ACCT [28]	BCAT [30]	CIS [34]	Mac-CAT-T [7]	U-Doc [50]
1. Decision-making situation regarding treatment options not a premise of the instrument?	X	X	X	X	(X)
2. Scope of application not limited to a specific illness or group of people?	✓	✓	X	✓	✓
3. Assessment of aspects other than the four abilities?	✓	(✓)	✓	X	✓
4. Inclusion of information about assessors?	X	X	X	X	✓

## Discussion

The main findings of our review are, on the one hand, the existence of a large number of instruments for assessing capacity to consent to treatment which are heterogeneous in several respects, including the operationalization of established criteria for decisional capacity. On the other hand, no instrument could be identified that could be applied to assessing decisional capacity in the context of requests for assisted suicide without any limitations or without necessitating adjustments. Whether these limitations are relevant for the conceptualization of decisional capacity the instruments are based on itself or whether applying these instruments to requests for assisted suicide would only necessitate adjusting items to this specific context – similarly to specific decision-making situations about treatment – remains open for discussion. For the most part, the instruments identified show deficits regarding psychometric properties and quality. Data on interrater reliability is reported for most instruments, but there is often a lack of further information on the reliability of these instruments. Additionally, given the known challenges when conceptualizing what constitutes a gold standard for validating instruments assessing decisional capacity, in order to test the validity, the assessment done using the instrument was often compared with the judgment of experts [20] or established instruments, such as the Mini-Mental State Examination (MMSE). Comparison with the MMSE has been criticized [21, 22] as it is not suitable for the assessment of decisional capacity [23, 58].

When comparing the instruments, it should be noted that different terms such as “capacity,” “competence” or “competency” are mentioned. This does not necessarily imply different underlying concepts as it could be due to linguistic differences. However, legislation on decisional capacity – and thus its conceptualization – varies between different countries, which could be reflected in the terminology. Despite these potential differences, the majority of the instruments are based on the four abilities model [7]. However, the approach to operationalizing these four abilities can differ considerably. Distinguishing whether the different ways of operationalizing abilities affect the conceptualization of decisional capacity itself or can rather be seen as heterogenous interpretations of the concept went beyond this project. Other differences between the instruments relate to an extension of the concept of decisional capacity beyond the four abilities to include, for example, values or emotions as a separate part of the assessment, as is the case with the U-Doc. These and other extensions regarding the conceptualization and operationalization of decisional capacity take into account the criticism of a more cognitive-oriented capacity assessment [8, 15].

Another reason for the heterogeneity of the instruments is the assessment of aspects that relate to the process of testing decisional capacity. A notable example is the reference to factors that could have an influence on the capacity judgment of the assessors by the U-Doc form. In view of empirical data on the potential impact of individual moral values on the assessment of decisional capacity [10, 18], the disclosure of such factors could be a useful impetus for the assessors’ reflection process.

Regarding the assessment of decisional capacity in the context of requests for assisted suicide, when analyzing the items, we were unable to find an instrument during our research that could be directly applied to this situation. Six instruments fulfilled the inclusion criteria for further analysis since they assess all four abilities outlined by Grisso and Appelbaum [7] and can be applied to the decision at hand. All five instruments that could be analyzed are based on the premise that the person being assessed has to make a decision regarding treatment options; however, items of both the U-Doc and the BCAT can be adapted to other healthcare decision-making situations and, in the case of the U-Doc, also to situations in which no illness is present. Requests for assisted suicide are not always made in the context of an illness, but can also be made, for example, by elderly people who do not wish to continue living [59, 60]. Since assisted suicide is legal in some countries even in the absence of illness, the remaining instruments are currently not applicable for such situations.

Furthermore, it remains unclear which kind of requirements should be set for decisional capacity in the context of assisted suicide, including whether there could be other approaches to operationalizing decisional capacity and whether these requirements could be similar to those for decision-making situations regarding treatment options as requests for assisted suicide differ from established medical decision-making situations. Against the background of interpretations of decisional capacity as a relational ability according to which requirements for decisional capacity should be adapted relative to the risks associated with a decision [61, 62], it thus also remains unclear whether instruments developed for assessing capacity to consent to treatment can be applied in principle to the context of assisted suicide. Current efforts to create an instrument for assessing decisional capacity in the context of requests for assisted suicide by the interdisciplinary German research group Forschungsnetzwerk Suizidassistenz (https://www.forschungsnetzwerk-suizidassistenz.de) funded by the German Research Foundation, for example, also take such aspects into consideration.

## Limitations

One limitation of the search is the possibility of not including thematically relevant articles or instruments even if predefined inclusion criteria were met. In order to minimize this risk, the research was conducted systematically, and all articles were screened independently by two researchers. As there was only a small number of articles identified in the course of our search update and due to limited resources, the quality of the included articles was not appraised. For the analysis, one limitation consists of the focus on the four abilities model as outlined by Grisso and Appelbaum [7]. This model was chosen as it is widely used, but other influential frameworks for decisional capacity, such as the Mental Capacity Act 2005 [63] could also provide a relevant approach to assessing capacity in the context of requests for assisted suicide. Due to the lack of data on the practice of assessing decisional capacity, the development of the questions for the structured analysis of the applicability of the instruments to assisted suicide was based primarily on theoretical premises and only to a limited extent on (empirical) studies of practice.

## Conclusions

In view of the emphasis on decisional capacity as an essential prerequisite for assisted suicide and studies that point to different approaches to assessing decisional capacity, assessment instruments that were specifically developed for the context of requests for assisted suicide could be helpful in establishing a more structured procedure. This applies in particular when these instruments are based on and combined with other important approaches, such as further developing a framework for assessing decisional capacity in the context of requests for assisted suicide or the training of potential assessors. Against the background of the heterogeneity shown in this study regarding the conceptualization and operationalization of decisional capacity and the interrelation of both aspects as well as the lack of instruments without deficits concerning psychometric properties that can also be used in the context of requests for assisted suicide, however, considerable research is required. Considering the need to operationalize a normative concept empirically, this should be carried out on an interdisciplinary basis. Additionally, it remains up for debate whether other ways of supporting the process of assessing decisional capacity in the context of requests for assisted suicide, such as developing guidelines or questionnaires and other more open tools, could also be feasible.

## Supporting information

10.1192/j.eurpsy.2025.10041.sm001 Kupsch et al. supplementary material Kupsch et al. supplementary material

## Data Availability

As no new data were created in the course of this review, data sharing is not applicable.

## Supplementary material

The supplementary material for this article can be found at http://doi.org/10.1192/j.eurpsy.2025.10041.
