# Supplementary material for: Assessment of decisional capacity. A systematic review and analysis of instruments regarding their applicability to requests for assisted suicide
Source: Eur Psychiatry. 2025 Jul 8;68(1):e91. doi: 10.1192/j.eurpsy.2025.10041 (PMC12260724; doi:10.1192/j.eurpsy.2025.10041)
Supplement: Kupsch et al. supplementary material [file S0924933825100412sup001.docx]

**Supplemental material to “Assessment of decisional capacity. A systematic review and analysis of instruments regarding their applicability to requests for assisted suicide”**

**Appendix 1: Systematic research**

**1. First search: October 1, 2022**

**1.1 MEDLINE (Ovid)**

| **#** | **Search terms** | **Results** |
| --- | --- | --- |
| 1 | informed consent.ab,sh,ti. | 69208 |
| 2 | (decision* adj3 capacit*).ab,ti. | 2340 |
| 3 | (capacit* adj3 consent).ab,ti. | 874 |
| 4 | disclosed information.ab,ti. | 85 |
| 5 | (information adj3 reasoning).ab,ti. | 275 |
| 6 | (express* adj3 choice).ab,ti. | 583 |
| 7 | consent form?.ab,ti. | 3525 |
| 8 | mental capacity.ab,sh,ti. | 1168 |
| 9 | mental competen*.ab,ti. | 167 |
| 10 | mental competency/ | 8581 |
| 11 | mental incompeten*.ab,ti. | 52 |
| 12 | decision making/ or decision making.ab,ti. | 234454 |
| 13 | (macarthur adj3 tool).ab,ti. | 170 |
| 14 | maccat.ab,ti. | 138 |
| 15 | consent questionnaire.ab,ti. | 32 |
| 16 | deaconess informed.ab,ti. | 2 |
| 17 | two part consent.ab,ti. | 4 |
| 18 | (california adj3 appreciation).ab,ti. | 4 |
| 19 | vignette method?.ab,ti. | 45 |
| 20 | informed consent survey.ab,ti. | 4 |
| 21 | competency interview schedule.ab,ti. | 2 |
| 22 | assessment of consent capacity for treatment.ab,ti. | 1 |
| 23 | Hopemont capacity assessment interview.ab,ti. | 1 |
| 24 | aid to capacity evaluation.ab,ti. | 7 |
| 25 | direct assessment of decision making capacity.ab,ti. | 1 |
| 26 | competency questionnaire.ab,ti. | 49 |
| 27 | SICIATRI.ab,ti. | 3 |
| 28 | structured interview for competenc*.ab,ti. | 3 |
| 29 | (hopkins adj2 assessment).ab,ti. | 25 |
| 30 | brief informed consent.ab,ti. | 1 |
| 31 | capacity assessment.ab,ti. | 643 |
| 32 | capacity to consent to treatment.ab,ti. | 117 |
| 33 | ("assessment tool" or "assessment tool").ab,ti. | 21754 |
| 34 | ("assessment instrument" or "assessment instruments").ab,ti. | 6100 |
| 35 | exp Psychological Tests/ | 341082 |
| 36 | ("competency assessment" or "competence assessment").ti,ab. | 1448 |
| 37 | (scale or inventory or assessment).ab,ti. | 393040 |
| 38 | 1 or 2 or 3 or 4 or 5 or 6 or 7 or 8 or 9 or 10 or 11 or 12 | 304186 |
| 39 | 13 or 14 or 15 or 16 or 17 or 18 or 19 or 20 or 21 or 22 or 23 or 24 or 25 or 26 or 27 or 28 or 29 or 30 | 372 |
| 40 | 31 or 32 or 33 or 34 or 35 or 36 or 37 | 366816 |
| 41 | 39 or 40 | 366958 |
| 42 | 38 and 41 | 8866 |
| 43 | limit 42 to yr="2018 -Current" | 1936 |

**1.2 Web of Science**

| **#** | **Search terms** | **Results** |
| --- | --- | --- |
| 1 | TS=(Informed consent) | 48611 |
| 2 | (TS=("decision-making capacity")) OR TS=("decision making capacity") | 1571 |
| 3 | TS=(capacity for consent) | 3486 |
| 4 | TS=("express* choice") | 37274 |
| 5 | TS=(consent form?) | 3604 |
| 6 | TS=(Mental capacity) | 16496 |
| 7 | TS=(Mental competenc*) | 10752 |
| 8 | TS=(mental incompeten*) | 594 |
| 9 | TS=(capacity assessment) | 70864 |
| 10 | TS=("capacity to consent to treatment") | 102 |
| 11 | (TS=("assessment tool")) OR TS=("assessment tools") | 43369 |
| 12 | (TS=("assessment instrument")) OR TS=("assessment instruments") | 8043 |
| 13 | (TS=("competency assessment")) OR TS=("competence assessment") | 1907 |
| 14 | #9 OR #11 OR #12 OR #13 OR #10 | 120847 |
| 15 | #2 OR #1 OR #3 OR #4 OR #5 OR #6 OR #7 OR #8 | 115142 |
| 16 | #14 AND #15 | 4156 |
| 17 | (#16), Timespan: 2018-01-01 to 2022-10-01 | 1764 |

**1.3 Cochrane Central Register of Controlled Trials (CENTRAL)**

| **#** | **Search terms** | **Results** |
| --- | --- | --- |
| 1 | MeSH descriptor: [Mental Competency] this term only | 85 |
| 2 | Informed Consent | 83169 |
| 3 | “decision-making capacity” | 75 |
| 4 | express choice | 617 |
| 5 | “consent form?” | 12552 |
| 6 | mental capacity | 4076 |
| 7 | mental competenc* | 1845 |
| 8 | mental incompetenc* | 43 |
| 9 | MeSH descriptor: [Decision Making] this term only | 2341 |
| 10 | “capacity assessment“ | 82 |
| 11 | “capacity to consent to treatment” | 5 |
| 12 | “assessment tool” OR “assessment tools” | 7857 |
| 13 | “assessment instrument” OR “assessment instruments” | 794 |
| 14 | MeSH descriptor: [Psychological Tests] explode all trees | 23078 |
| 15 | “competency assessment” OR “competence assessment” | 160 |
| 16 | #1 OR #2 OR #3 OR #4 OR #5 OR #6 OR #7 OR #8 OR #9 | 93846 |
| 17 | #10 OR #11 OR #12 OR #13 OR #14 OR #15 | 9749 |
| 18 | #16 AND #17 | 1188 |
| 19 | #16 AND #17, with Publication Year from 2018 to 2022, with Cochrane Library publication date from Jan 2018 to Oct 2022, in Trials | 267 |
| 20 | #19, only published, no trial registers | 84 |

**1.4 CINAHL**

| **#** | **Search terms** | **Results** |
| --- | --- | --- |
| 1 | decision making capacity | 1329 |
| 2 | capacity for consent | 857 |
| 3 | mental competency | 2222 |
| 4 | mental capacity | 1895 |
| 5 | mental incompeten* | 28 |
| 6 | "capacity to consent to treatment" | 64 |
| 7 | assessment tools or assessment method or assessing | 347453 |
| 8 | assessment instrument* | 7758 |
| 9 | "competency assessment" | 6754 |
| 10 | "competence assessment" | 3711 |
| 11 | #1 OR #2 OR #3 OR #4 OR #5 OR #6 | 3657 |
| 12 | (MM "Clinical Assessment Tools") | 16993 |
| 13 | #7 OR #8 OR #9 OR #10 OR #12 | 357019 |
| 14 | #11 AND #13 | 518 |
| 15 | #11 AND #13, Eingrenzungen - Erscheinungsdatum: 20180101-20221231 | 156 |

**1.5 PsycINFO**

| **#** | **Search terms** | **Results** |
| --- | --- | --- |
| 1 | (macarthur adj3 tool) | 98 |
| 2 | maccat | 53 |
| 3 | consent questionnaire | 12 |
| 4 | deaconess informed | 6 |
| 5 | two part consent | 21 |
| 6 | (california adj3 appreciation) | 21 |
| 7 | vignette method? | 3123 |
| 8 | informed consent survey | 23 |
| 9 | competency interview schedule | 92 |
| 10 | assessment of consent capacity for treatment | 31 |
| 11 | Hopemont capacity assessment interview | 12 |
| 12 | aid to capacity evaluation | 11 |
| 13 | competency questionnaire | 13 |
| 14 | SICIATRI | 7 |
| 15 | structured interview for competenc* | 9 |
| 16 | (hopkins adj2 assessment) | 21 |
| 17 | brief informed consent | 19 |
| 18 | (competency adj3 interview) | 11 |
| 19 | (Hopemont adj4 interview) | 19 |
| 20 | testing/ | 13001 |
| 21 | decision-making | 31555 |
| 22 | informed consent | 40121 |
| 23 | voluntary consent | 4444 |
| 24 | (decision* adj1 capacit*) | 8369 |
| 25 | OR 1-19 | 2222 |
| 26 | 20 OR 25 | 13052 |
| 27 | OR 21-24 | 82461 |
| 28 | 26 AND 27 | 4159 |
| 29 | 28 (Filter 2018-2022) | 324 |

**2. Second search: March 15, 2024**

**2.1 MEDLINE (Ovid)**

| **#** | **Search terms** | **Results** |
| --- | --- | --- |
| 1 | informed consent.ab,sh,ti. | 73406 |
| 2 | (decision* adj3 capacit*).ab,ti. | 2595 |
| 3 | (capacit* adj3 consent).ab,ti. | 941 |
| 4 | disclosed information.ab,ti. | 89 |
| 5 | (information adj3 reasoning).ab,ti. | 299 |
| 6 | (express* adj3 choice).ab,ti. | 618 |
| 7 | consent form?.ab,ti. | 3961 |
| 8 | mental capacity.ab,sh,ti. | 1234 |
| 9 | mental competen*.ab,ti. | 171 |
| 10 | mental competency/ | 8640 |
| 11 | mental incompeten*.ab,ti. | 52 |
| 12 | decision making/ or decision making.ab,ti. | 262657 |
| 13 | (macarthur adj3 tool).ab,ti. | 178 |
| 14 | maccat.ab,ti. | 142 |
| 15 | consent questionnaire.ab,ti. | 35 |
| 16 | deaconess informed.ab,ti. | 2 |
| 17 | two part consent.ab,ti. | 5 |
| 18 | (california adj3 appreciation).ab,ti. | 4 |
| 19 | vignette method?.ab,ti. | 49 |
| 20 | informed consent survey.ab,ti. | 4 |
| 21 | competency interview schedule.ab,ti. | 2 |
| 22 | assessment of consent capacity for treatment.ab,ti. | 1 |
| 23 | Hopemont capacity assessment interview.ab,ti. | 1 |
| 24 | aid to capacity evaluation.ab,ti. | 7 |
| 25 | direct assessment of decision making capacity.ab,ti. | 1 |
| 26 | competency questionnaire.ab,ti. | 65 |
| 27 | SICIATRI.ab,ti. | 4 |
| 28 | structured interview for competenc*.ab,ti. | 4 |
| 29 | (hopkins adj2 assessment).ab,ti. | 27 |
| 30 | brief informed consent.ab,ti. | 1 |
| 31 | capacity assessment.ab,ti. | 750 |
| 32 | capacity to consent to treatment.ab,ti. | 125 |
| 33 | ("assessment tool" or "assessment tool").ab,ti. | 26023 |
| 34 | ("assessment instrument" or "assessment instruments").ab,ti. | 6617 |
| 35 | exp Psychological Tests/ | 349766 |
| 36 | ("competency assessment" or "competence assessment").ti,ab. | 1628 |
| 37 | (scale or inventory or assessment).ab,ti. | 2231807 |
| 38 | 1 or 2 or 3 or 4 or 5 or 6 or 7 or 8 or 9 or 10 or 11 or 12 | 336611 |
| 39 | 13 or 14 or 15 or 16 or 17 or 18 or 19 or 20 or 21 or 22 or 23 or 24 or 25 or 26 or 27 or 28 or 29 or 30 | 405 |
| 40 | 31 or 32 or 33 or 34 or 35 or 36 or 37 | 380231 |
| 41 | 39 or 40 | 380397 |
| 42 | 38 and 41 | 9301 |
| 48 | limit 42 to dt=20221001-20240315 | 417 |

**2.2 Web of Science**

| **#** | **Search terms** | **Results** |
| --- | --- | --- |
| 1 | TS=(Informed consent) | 52566 |
| 2 | (TS=("decision-making capacity")) OR TS=("decision making capacity") | 1748 |
| 3 | TS=(capacity for consent) | 3876 |
| 4 | TS=("express* choice") | 59 |
| 5 | TS=(Mental capacity) | 18767 |
| 6 | TS=(Mental competenc*) | 12156 |
| 7 | TS=(mental incompeten*) | 621 |
| 8 | TS=(capacity assessment) | 81893 |
| 9 | TS=("capacity to consent to treatment") | 111 |
| 10 | (TS=("assessment tool")) OR TS=("assessment tools") | 50919 |
| 11 | (TS=("assessment instrument")) OR TS=("assessment instruments") | 8623 |
| 12 | (TS=("competency assessment")) OR TS=("competence assessment") | 2127 |
| 13 | TS=(consent form?) | 3951 |
| 14 | #1 OR #2 OR #3 OR #4 OR #5 OR #6 OR #7 OR #13 | 86126 |
| 15 | #8 OR #9 OR #10 OR #11 OR #12 | 139757 |
| 16 | #14 AND #15 | 4589 |
| 17 | (#16), Timespan: 2022-10-01 to 2024-03-15 | 611 |

**2.3 Cochrane Central Register of Controlled Trials (CENTRAL)**

| **#** | **Search terms** | **Results** |
| --- | --- | --- |
| 1 | MeSH descriptor: [Mental Competency] this term only | 124 |
| 2 | Informed Consent | 100238 |
| 3 | “decision-making capacity” | 79 |
| 4 | express choice | 631 |
| 5 | consent NEXT form? | 18530 |
| 6 | mental capacity | 4791 |
| 7 | mental competenc* | 2114 |
| 8 | mental incompetenc* | 48 |
| 9 | MeSH descriptor: [Decision Making] this term only | 3410 |
| 10 | “capacity assessment“ | 96 |
| 11 | “capacity to consent to treatment” | 6 |
| 12 | “assessment tool” OR “assessment tools” | 5171 |
| 13 | “assessment instrument” OR “assessment instruments” | 878 |
| 14 | MeSH descriptor: [Psychological Tests] explode all trees | 27744 |
| 15 | “competency assessment” OR “competence assessment” | 182 |
| 16 | #1 OR #2 OR #3 OR #4 OR #5 OR #6 OR #7 OR #8 OR #9 | 113626 |
| 17 | #10 OR #11 OR #12 OR #13 OR #14 OR #15 | 33747 |
| 18 | #16 AND #17 | 1538 |
| 19 | #16 AND #17, with Publication Year from 2022 to present, with Cochrane Library publication date from Oct 2022 to present, in Trials | 83 |
| 20 | #19, only published, no trial registers | 21 |

**2.4 CINAHL**

| **#** | **Search terms** | **Results** |
| --- | --- | --- |
| 1 | decision making capacity | 1364 |
| 2 | capacity for consent | 860 |
| 3 | mental competence | 2336 |
| 4 | mental capacity | 1891 |
| 5 | mental incompeten* | 26 |
| 6 | "capacity to consent to treatment" | 68 |
| 7 | assessment tools or assessment method or assessing | 361077 |
| 8 | assessment instrument* | 8125 |
| 9 | "competency assessment" | 7494 |
| 10 | "competence assessment" | 4225 |
| 11 | (MM "Clinical Assessment Tools") | 18581 |
| 12 | #1 OR #2 OR #3 OR #4 OR #5 OR #6 | 5390 |
| 13 | #7 OR #8 OR #9 OR #10 OR #11 | 371388 |
| 14 | #11 AND #13 | 719 |
| 15 | #11 AND #13, Eingrenzungen - Erscheinungsdatum: 20221001- | 39 |

**2.5 PsycINFO**

| **#** | **Search terms** | **Results** |
| --- | --- | --- |
| 1 | (macarthur adj3 tool) | 13 |
| 2 | maccat | 168 |
| 3 | consent questionnaire | 453 |
| 4 | deaconess informed | 2 |
| 5 | two part consent | 5 |
| 6 | (california adj3 appreciation) | 27 |
| 7 | vignette method? | 570 |
| 8 | informed consent survey | 192 |
| 9 | competency interview schedule | 14 |
| 10 | assessment of consent capacity for treatment | 59 |
| 11 | Hopemont capacity assessment interview | 26 |
| 12 | aid to capacity evaluation | 25 |
| 13 | competency questionnaire | 410 |
| 14 | SICIATRI | 2 |
| 15 | structured interview for competenc* | 224 |
| 16 | (hopkins adj2 assessment) | 95 |
| 17 | brief informed consent | 25 |
| 18 | (competency adj3 interview) | 51 |
| 19 | (Hopemont adj4 interview) | 26 |
| 20 | MM ("testing") | 6763 |
| 21 | decision-making | 158203 |
| 22 | informed consent | 13147 |
| 23 | voluntary consent | 273 |
| 24 | (decision* adj1 capacit*) | 21 |
| 25 | OR 1-19 | 4985 |
| 26 | 20 OR 25 | 11746 |
| 27 | OR 21-24 | 169034 |
| 28 | 26 AND 27 | 997 |
| 29 | 28 (Limiters - Publication Date: 20221001-) | 66 |
